# Comment on: Longitudinal risk of serious infections in patients with inflammatory arthritis on immunomodulating therapy compared to controls

**DOI:** 10.1093/rap/rkaf068

**Published:** 2025-06-05

**Authors:** Rebecca Parry

**Affiliations:** Emergency Medicine, The Grange University Hospital, Cwmbran, UK

Dear Editor, I read *Longitudinal risk of serious infections in patients with inflammatory arthritis on immunomodulating therapy compared to controls* by Christensen *et al.* [1] which I think is a really important study as immunomodulating therapies are increasingly being used not only in inflammatory arthritis but in other conditions. It is therefore necessary to evaluate potential complications of these drugs, in particular infection risk, to ensure the appropriate initiation of these drugs and patient education surrounding the potential risks.

The article raised several questions which I think are important to address. Does the study include data with the breakdown of the infection rate for each specific drug? The article alluded to rituximab causing higher rates of infection within the discussion regarding limitations of the study; however, there is data suggesting stable rates of infection for this cohort of patients [2]. It would be useful to see this breakdown as gaining more data comparing therapies and the infection rate could be influential to prescribers when choosing therapies to initiate.

Does the study have data on the type of serious infections? This again would be useful to see if there is a trend, as increased data in this area would help educate prescribers and patients, and perhaps symptoms would be identified and treated sooner if there were a caution regarding risk of certain infections. This would help prevent hospital admissions.

Finally, does the data include patient mortality alongside the morbidity of serious infection? Again this data would be useful, especially when compared to each immunomodulating agent, as this may affect the decision of prescribers.

## Data Availability

No new data was generated or analysed in support of this article.
